# A Recommendation System for Trigger–Action Programming Rules via Graph Contrastive Learning

**DOI:** 10.3390/s24186151

**Published:** 2024-09-23

**Authors:** Zhejun Kuang, Xingbo Xiong, Gang Wu, Feng Wang, Jian Zhao, Dawen Sun

**Affiliations:** 1 College of Computer Science and Technology, Changchun University, Changchun 130022, China; kuangzhejun@ccu.edu.cn (Z.K.); 231501519@mails.ccu.edu.cn (X.X.); zhaojian@ccu.edu.cn (J.Z.); 2 Jilin Provincial Key Laboratory of Human Health Status Identification Function & Enhancement, Changchun 130022, China; 3 Key Laboratory of Intelligent Rehabilitation and Barrier-Free for the Disabled, Changchun University, Ministry of Education, Changchun 130022, China; 4 College of Computer Science and Technology, Jilin University, Changchun 130012, China; wugang17@mails.jlu.edu.cn (G.W.); wangfeng12@mails.jlu.edu.cn (F.W.)

**Keywords:** Internet of Things, trigger–action programming, rule recommendation, graph contrastive learning

## Abstract

Trigger–action programming (TAP) enables users to automate Internet of Things (IoT) devices by creating rules such as “IF Device1.TriggerState is triggered, THEN Device2.ActionState is executed”. As the number of IoT devices grows, the combination space between the functions provided by devices expands, making manual rule creation time-consuming for end-users. Existing TAP recommendation systems enhance the efficiency of rule discovery but face two primary issues: they ignore the association of rules between users and fail to model collaborative information among users. To address these issues, this article proposes a graph contrastive learning-based recommendation system for TAP rules, named GCL4TAP. In GCL4TAP, we first devise a data partitioning method called DATA2DIV, which establishes cross-user rule relationships and is represented by a user–rule bipartite graph. Then, we design a user–user graph to model the similarities among users based on the categories and quantities of devices that they own. Finally, these graphs are converted into low-dimensional vector representations of users and rules using graph contrastive learning techniques. Extensive experiments conducted on a real-world smart home dataset demonstrate the superior performance of GCL4TAP compared to other state-of-the-art methods.

## 1. Introduction

**Figure 1:**
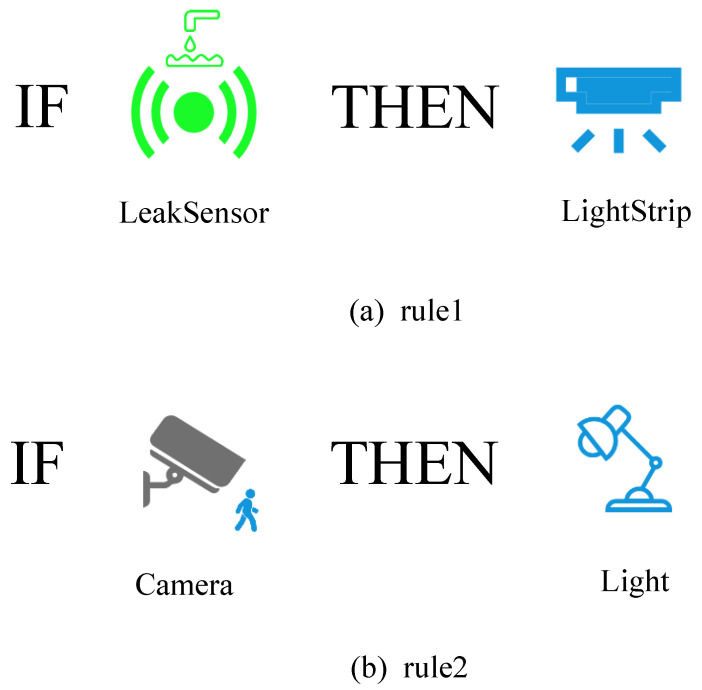
The structure of TAP rules: (**a**) Rule: “If the LeakSensor detects a leak, then the LightStrip’s color blinks”. (**b**) Rule: “If the Camera detects a person, then turn on the Light”.

With the advent of the 5G era, an increasing number of Internet of Things (IoT) devices and online services are interconnecting with each other, enabling unprecedented automation and efficiency. In this context, trigger–action programming (TAP) has become a key approach enabling users to define automation rules to automate their devices [1]. These rules typically follow the “IF Device1.TriggerState is triggered, THEN Device2.ActionState is executed” format, which simplifies the management of the complex IoT ecosystem. Using this programming approach, users can easily configure interactions between home devices. For instance, Figure 1 demonstrates two rules: rule1 indicates that when the trigger device LeakSensor detects a leak, it will trigger the execution device LightStrip to blink its color; rule2 indicates that when the trigger device camera detects a person, it will trigger the execution device Light to turn on the light. TAP not only improves the efficiency of collaboration between devices but also significantly enhances the convenience and comfort of users [2].

Although TAP offers many advantages, the rapid proliferation of IoT devices poses significant challenges. Firstly, the growing number of devices creates a vast combination space for the functions that they offer, making manual rule creation not only tedious but also impractical for most end-users. Each device can offer multiple triggers or actions. For example, a camera can trigger the function “detect motion” or execute the function “turn off notifications”. Therefore, effective recommendation systems are needed to help users to discover and configure automation rules efficiently. Secondly, the question of how to distinguish rules between different instances of the same type of device is also a challenge. For example, camera1 in the kitchen and camera2 in the corridor belong to different instance devices of the same type. They are, respectively, connected to light1 and light2, which are different instance devices of the same type. When camera1 detects a gas leak, light1 will be triggered to flash a color prompt; when camera2 detects a person, light2 will be triggered to turn on the light. The issue of how to distinguish the rules between camera1 and light1 and the rules between camera2 and light2 represents an important problem. This complexity requires an intelligent recommendation system capable of analyzing the user’s needs and the device characteristics to accurately recommend appropriate rules.

Existing TAP recommendation systems have shown progress in facilitating rule discovery. Kim et al. [3] proposed the use of a node2vec approach to predict the corresponding execution devices based on user-specified trigger devices. Huang et al. [4] proposed a method called TAP-AHGNN to recommend feasible action services for user-specified trigger services to automatically complete the TAP rules. Some studies [5,6,7] have used natural language processing technology to learn users’ needs and recommend corresponding services based on user inputs. However, these methods do not consider the relationship between users and rules. Recently, Yao et al. [8] proposed the FedRule method, which constructs the rules used by each user into a graph, effectively distinguishing rules between different instances of devices of the same type. However, they did not use collaborative information among users, significantly reducing the performance of TAP rule recommendations.

In this article, firstly, to solve the problems faced by manually creating rules, we propose a TAP recommendation system based on graph contrastive learning, named GCL4TAP. Secondly, to distinguish the rules between different instance devices of the same type, we design a data partitioning method named DATA2DIV. Previous research has ignored the differences between different instance devices of the same type and assigned the same number to the devices of this type [9]. In our data partitioning method, we assign unique numbers to the different instance devices of each type, enabling us to effectively distinguish the rules between them. Thirdly, to address the problem whereby previous studies have not considered the association of rules between different users or the collaborative information among users [10], we first establish the linkage of rules across users and the similarity between users based on the categories and quantities of devices that they own. Then, we derive a low-dimensional vector representation of the users and rules through the graph contrastive learning technique. After fully considering the rule associations between different users and the collaboration information among users, our proposed GCL4TAP method not only improves the efficiency of rule discovery but also enables more accurate and personalized rule recommendations. Finally, we compare GCL4TAP with several methods on the Wyze dataset. The results show that our proposed GCL4TAP method outperforms all compared methods in providing personalized TAP rule recommendations. The contributions of this article are as follows.

1. We propose a TAP rule recommendation system based on graph contrastive learning, which aims to integrate the relationships between rules of different users and the similarity among users to learn low-dimensional vector representations of the users and rules, providing users with efficient and accurate TAP rule recommendations.
2. We propose a data partitioning method called DATA2DIV, to establish the associations of rules between users. By considering the category and number of devices owned by users, we design a user–user graph to represent the collaborative relationship among users.
3. We perform TAP rule recommendation on the Wyze dataset to test our proposed GCL4TAP framework. The results show that our framework effectively provides personalized TAP rule recommendations and outperforms the state-of-the-art compared methods.

The rest of this article is organized as follows. Section 2 reviews related works. Preliminaries and symbol definitions are described in Section 3. We give the technical details of the designed framework in Section 4. Section 5 presents our experiments. Furthermore, Section 6 concludes the article.

## 2. Related Work

In this section, we review some recent research on TAP. Then, we present the current state of research on TAP rule-based recommendation and graph contrastive learning methods used for recommendation.

### 2.1. TAP in IoT

There are a number of research works focusing on the automatic generation of TAP rules. Yusuf et al. [5] framed the TAP combination as a multi-classification task and proposed a deep learning method to generate structured TAP rules by predicting the text content in each slot. Zhang et al. [11] used symbolic execution and SAT-solving techniques to generate all possible rules related to the target action and ranked these rules for selection. Sunil et al. [12] connected various user-defined pairs of triggered actions and constructed extended “T-A-T-A” sequences. These synthesized long sequences were used to build N-metamodels for home automation. Corno et al. [13] proposed an end-user development platform called TAPrec, which not only recommends new trigger–action rules and automates their completion but also dynamically updates the recommended rules in real time. Zhang et al. [11] introduced an approach called Trac2tap, which automatically synthesizes TAP rules from traces (timestamped logs of sensor readings and manual device activations). Additionally, the authors developed a visualization interface to display the synthesized rules.

We notice a drawback in the above-mentioned studies, i.e., the information on the trigger devices and action devices is not taken into account while generating the TAP rules. In our proposed GCL4TAP approach, we incorporate this device information, which significantly enhances the performance of TAP rule recommendation.

### 2.2. Recommendations for TAP Rules

With the continuous development of communication technology, the number of smart devices/services has shown explosive growth. The combinations between triggers and actions have become increasingly numerous, and it is very difficult for a new end-user to express his/her needs through TAP. In this context, it is particularly important to provide effective TAP rule recommendations to end-users. A large number of researchers have been engaged in this area. Wu et al. [14] proposed a semantic-aware approach called RTAR to recommend trigger–action rules to users. Firstly, the authors modeled the relationships between IoT devices, services, triggers, and actions by designing a trigger–action knowledge graph. Secondly, they extracted features from this graph and trained a ranking model to learn how to recommend appropriate trigger–action rules. Corno et al. [13] developed an end-user development platform called TAPrec, which dynamically recommends trigger–action rule combinations. By leveraging hybrid and semantic recommendation algorithms, TAPrec can either suggest a new rule or auto-complete the action for an existing rule. The recommendations align with the user’s high-level intent, focusing on the rule’s ultimate purpose rather than low-level specifics like the manufacturer or brand. Forouzandeh et al. [15] introduced a method called UIFRS-HAN, which offers an excellent approach to personalized recommendations. This method leverages heterogeneous information networks (HIN) and a dual attention mechanism to capture the complex relationships between users and items, thereby providing personalized recommendations. Huang et al. [4] proposed a method called TAP-AHGNN to recommend feasible action services based on user-specified trigger services for the automation of TAP rules. This approach consists of the following steps: firstly, a heterogeneous TAP knowledge graph is constructed, from which five meta-paths are extracted to build a service neighborhood. Secondly, a multi-level attention-based heterogeneous graph convolution module is integrated to selectively aggregate neighborhood information, followed by a transformer-based fusion module to combine multiple types of feature information. With these integrated modules, the final service representation captures both semantic and structural information, resulting in better recommendation outcomes.

In conclusion, the research work mentioned above focuses on rule construction and recommendation from a semantic-aware perspective and does not analyze it from the perspective of device information. We believe that the generation of TAP rules is correlated with the device information possessed by the user, and, based on this assumption, we fuse the device information and propose the GCL4TAP method.

### 2.3. Graph Contrastive Learning

Graph neural networks have emerged as an effective method for collaborative filtering in recommendation systems. In recent years, numerous studies have incorporated contrastive learning into graph neural network-based recommendation systems to address the issue of label sparsity in self-supervised signals. SGL proposed by Wu et al. [16] and simGCL proposed by Yu et al. [17] perform data augmentation over graph structures and embeddings through random dropout operations. However, this random deactivation operation may cause the original structure and embedding to lose important information, which causes the sparsity problem to become more serious for inactive users. Jiang et al. [18] proposed an adaptive graph contrastive learning method called AdaGCL. The method performs data augmentation operations through two adaptive contrast view generators to better enhance the collaborative filtering paradigm. The authors created adaptive contrastive views by using two trainable view generators, i.e., a graph generation model and a graph denoising model, for the creation of adaptive contrast views through which the AdaGCL method can integrate additional high-quality training signals into the collaborative filtering paradigm to effectively mitigate the data sparsity problem. Jing et al. [19] proposed an adaptive data augmentation method for recommendation called GCARec. In this method, the importance of each edge is calculated using an attention mechanism, which determines whether an edge should be retained. Unimportant edges are perturbed through the same attention mechanism. The edges with high and low retention probabilities are sampled using Gumbel Softmax [20]. This process generates two views, and the model is optimized by maximizing the consistency between the views through an infoNCE loss function, which serves as the objective [21,22].

In this article, we propose the GCL4TAP method, which firstly reconstructs the original view using singular value decomposition. Then, a new view is formed from the reconstructed graph and used to calculate the contrastive loss with the original graph. Additionally, a user–user graph is constructed from the device information, and the graph is also used to calculate the contrastive loss with the original user graph. Finally, the two-part contrastive loss is added to the model’s objective function for joint multi-task training, ultimately improving the model’s recommendation performance.

## 3. Preliminaries

In this section, we present the preliminaries for TAP and graph contrastive learning. The commonly used notational definitions for GCL4TAP are shown in the Table 1 below.

### 3.1. Preliminaries for TAP

TAP is a very popular programming paradigm in the IoT and is based on trigger–action rules that automate the execution of services for web applications and IoT devices. The TAP programming paradigm does not require complex programming languages to write code, so it is highly suitable for non-professional end-users. Users can perform programming in the form of a set of trigger–action rules to automate IoT devices and online services according to their needs. There are already some well-established TAP websites and datasets, such as IFTTT (https://ifttt.com/, accessed on 30 June 2024), Zapier (https://zapier.com/, accessed on 30 June 2024), and Wyze (https://www.wyze.com/, accessed on 30 June 2024), where millions of trigger–action rules have already been created on these websites or well-established datasets. In this article, we focus on the rule data in the Wyze dataset. Figure 1 shows two example rules from the Wyze dataset.

The structure and composition of the rules in the Wyze dataset are shown in Table 2. Each rule consists of four parts: a trigger device, trigger state, action device, and action state in the form of “if something triggers, then perform an action”. The function in Table 2 represents the state of the device. For example, a rule in Figure 1 indicates that there is a LeakSensor and a LightStrip, which represent a trigger device and an action device, respectively. The whole rule can be described as “If the LeakSensor detects a leak, then the LightStrip blinks”.

### 3.2. Preliminaries for Graph Contrastive Learning

Graph contrastive learning is an emerging graph representation learning method that can improve the performance of graph neural networks. Below are some basic concepts and preliminaries of graph contrastive learning.

**Graph representation learning:** This is a class of methods that extract useful features from graph-structured data. By learning embedding representations of graph nodes, edges, and the overall graph, support can be provided for a variety of downstream tasks, such as node classification, link prediction, and graph classification. In this article, the graph convolution operation is chosen to aggregate the feature information of the nodes, and the formula for the aggregation of the feature information of the nodes in the *k*-th layer is shown below [23]:

(1) $$e_{u}^{k}=AGG\left(e_{u}^{k-1},e_{i}^{k-1}\right)$$

where AGG(·) is an aggregation function—the core of graph convolution—that considers the *k*-th layer’s representation of the target node and its neighbor nodes. $e_{u}^{k-1}$ and $e_{i}^{k-1}$, respectively, denote the embedding representations of user *u* and item *i* after the propagation of the (k−1)-th layers.

**Contrastive Learning:** This is a self-supervised learning method that learns a representation of the data by maximizing the similarity between positive sample pairs and minimizing the similarity between negative sample pairs. Positive sample pairs are pairs between the original data and their augmented versions (e.g., the representation of the same node under different perturbations), while negative sample pairs are pairs between different data (e.g., the representation of different nodes). In graph contrastive learning, these sample pairs can be different views of the graph, different neighborhoods of nodes [24], etc.

**Contrastive loss function:** The most commonly used is the InfoNCE-based loss function, which aims to maximize the similarity of positive sample pairs while minimizing the similarity of negative sample pairs. The specific formula is shown below:

(2) $$L_{gcl}=\sum_{i=0}^{I}-\log\frac{\exp\left(s\left(e_{i},e_{i}^{a}\right)/t\right)}{\sum_{i\prime =0}^{I}\exp\left(s\left(e_{i},e_{i\prime }^{a}\right)/t\right)}$$

where s(·) and *t* denote the cosine similarity and temperature parameters, respectively. $e_{i}$ denotes the embedding representation of the original view and $e_{i}^{a}$ denotes the embedding representation of the augmented view.

## 4. GCL4TAP

**Figure 2:**
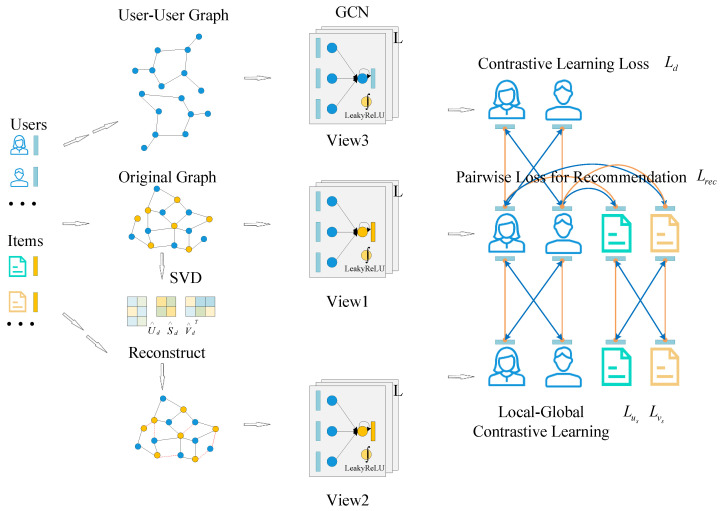
The overall structure of GCL4TAP.

In this section, we first introduce the proposed DATA2DIV data partitioning method. Then, we introduce our proposed GCL4TAP framework in detail. GCL4TAP is an approach based on the graph contrastive learning paradigm, and its overall framework is shown in Figure 2. The model consists of three parts. The top part is constructed from the similarity of user devices, and a user–user graph is constructed for later graph contrastive learning by calculating the cosine similarity between users and user-owned devices. The middle part aims to extract the local dependencies of users and items in the original data, which are used to preserve the local features of the original data. The lower part seeks to reconstruct the original data through the augmentation method of singular value decomposition and then learn the synergistic relationships between users and items from a global perspective to generate a new augmented contrastive view.

### 4.1. DATA2DIV

**Figure 3:**
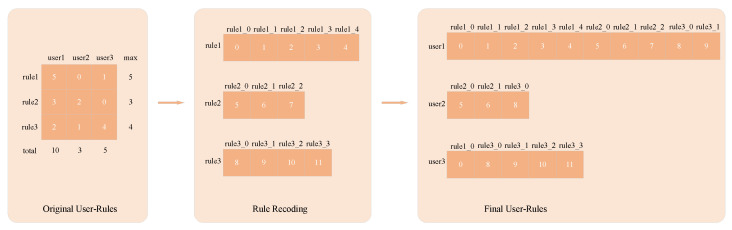
An example of DATA2DIV.

For multiple-instance devices under the same type owned by a user, the connection rules are different. For example, camera1 and camera2 belong to the same type of Camera, and light1 and light2 belong to the same type of Light. camera1 is installed in the hallway and connected to light1, which is used to trigger light1 to turn on when someone is detected to have passed through; camera2 is installed at the front door and connected to the warning light, which triggers light2 to blink when someone is detected entering. The numbering of the different rules connected to different instances of devices of different types owned by a user is a complex issue. Previous studies regard the rules connected to Camera and Light devices as the same types of rules, in which case, as shown in the original user-rules in Figure 3, the five specific rules contained in rule1, namely $rule1_{\_0},rule1_{\_1},rule1_{\_2},rule1_{\_3},rule1_{\_4}$, are counted as rule1. This does not effectively number the connection rules between specific instances. Here, to number the different instance device connection rules, we propose the DATA2DIV method.

An example of data partitioning using the DATA2DIV method is shown in Figure 3. Firstly, we count the number of rules between various device types that each user has, i.e., the original user-rules part of Figure 3, and, from this matrix, it is easy to derive the maximum number of each rule. Secondly, we assign a new virtual number to the specific rules under each rule according to the maximum number obtained (e.g., rule1 contains five specific rules, $rule1_{\_0},rule1_{\_1},rule1_{\_2},rule1_{\_3},rule1_{\_4}$, i.e., the connection rules between different instances of devices of the same type), as shown in Figure 3 in the rule recoding part. Finally, a new user–rule matrix is generated based on the newly assigned number, which is used to store the information of the connection rules between different instance devices under the same type owned by each user.

### 4.2. Details of GCL4TAP

#### 4.2.1. Local Graph Dependency Modeling

In our proposed TAP recommendation method, we aim to recommend the appropriate TAP rules to the user, so we refer to collaborative filtering and utilize the form of “user–items” to store our raw data information, where “user” denotes the user and “items” denotes the TAP rules owned by the user. Here, we use $u_{i}$ to denote the *i*-th user; we use $v_{j}$ to denote the *j*-th TAP rule. For each user and TAP rule, we represent it with an embedding vector: $e_{u_{i}}$, $e_{v_{j}}$$\in R^{D}$, where *D* denotes the size of the embedding vector. For the full set of users and rules, we can denote them as $E_{u}\in R^{m\times D}$, $E_{v}\in R^{n\times D}$, where *m* and *n* denote the number of users and the number of TAP rules, respectively. Then, we employ a two-layer GCN to aggregate the domain information of each node. The aggregation process at the *k*-th layer is shown below:

(3) $${\hat{e}}_{u_{i,k}}=\sigma \left(p\left({\hat{A}}_{i,:}\right)\cdot E_{v_{k-1}}\right),{\hat{e}}_{v_{j,k}}=\sigma \left(p\left({\hat{A}}_{:,j}\right)\cdot E_{u_{k-1}}\right)$$

where ${\hat{e}}_{u_{i,k}}$, ${\hat{e}}_{v_{j,k}}$ denote the aggregated embedding representations of user $u_{i}$ and rule $v_{j}$ at the *k*-th layer; $\hat{A}$ denotes the normalized adjacency matrix; p(·) denotes the random edge loss of the adjacency matrix, which aims to alleviate the overfitting problem; and σ(·) denotes the activation function.

The final embedding representation of user $u_{i}$ and rule $v_{j}$ is the sum of all layer embedding representations:

(4) $$e_{u_{i}}=\sum_{k=0}^{K}{\hat{e}}_{u_{i,k}},e_{v_{j}}=\sum_{k=0}^{K}{\hat{e}}_{v_{j,k}}$$

where $e_{u_{i}}$, $e_{v_{j}}$ denote the final embedding representations of user $u_{i}$ and rule $v_{j}$, respectively. Then, the inner product of the final embedding representations of user $u_{i}$ and rule $v_{j}$ is applied as a result of predicting the user’s preference for the rule:

(5) $${\hat{y}}_{i,j}=e_{u_{i}}^{T}e_{v_{j}}$$

where ${\hat{y}}_{i,j}$ denotes the result of predicting the user’s preference for the rule.

#### 4.2.2. Global Collaborative Relation Learning

In our proposed GCL4TAP method, an SVD mechanism is implemented, which aims to extract important collaborative filtering signals from a global perspective to enhance the performance of TAP rule recommendation. Specifically, we perform SVD on the normalized adjacency matrix $\hat{A}$, i.e., $\hat{A}=USV^{T}$, where *U* denotes the m×m orthogonal matrix, which is the eigenvector of the correlation matrix between the rows of the normalized adjacency matrix $\hat{A}$; *V* denotes the n×n orthogonal matrix, which is the eigenvector of the correlation matrix between the columns of the normalized adjacency matrix $\hat{A}$; and *S* is the m×n diagonal matrix, which is used to store the singular values of the normalized adjacency matrix $\hat{A}$. The maximum singular value is generally related to the principal components of the matrix. Therefore, we reconstruct the normalized adjacency matrix by keeping the top *d* largest singular values in the list of singular values, i.e., $\hat{A}=U_{d}S_{d}V_{d}^{T}$, where $U_{d}\in R^{m\times d}$, $V_{d}\in R^{n\times d}$ represent the top *d* rows of *U* and *V*, respectively; $S_{d}\in R^{d\times d}$ denotes the diagonal matrix corresponding to the top *d* largest singular values. The benefit of performing SVD is that this highlights the main components of the graph by identifying the user preferences in the user–item interaction graph, while the reconstructed new graph preserves the global co-signal by considering each user–item pair. We perform message propagation at each layer of the user–item interaction graph reconstructed by SVD:

(6) $${\hat{e}}_{u_{i,k}}^{R}=\sigma \left(p\left({\hat{A}}_{i,:}\right)\cdot E_{v_{k-1}}\right),{\hat{e}}_{v_{j,k}}^{R}=\sigma \left(p\left({\hat{A}}_{:,j}\right)\cdot E_{u_{k-1}}\right)$$

Performing exact SVD on large matrices is very costly, so we use the stochastic SVD algorithm, the core idea of which is to approximate the range of the input matrix with a low-rank orthogonal matrix and then perform SVD on this low-rank matrix:

(7) $${\hat{U}}_{d},{\hat{S}}_{d},{\hat{V}}_{d}^{T}=SVD\left(\hat{A},d\right),{\hat{A}}_{SVD}={\hat{U}}_{d}{\hat{S}}_{d}{\hat{V}}_{d}^{T}$$

where *d* is the rank required for matrix factorization; ${\hat{U}}_{d}\in R^{m\times d}$, ${\hat{V}}_{d}\in R^{n\times d}$, ${\hat{S}}_{d}\in R^{d\times d}$ denote the approximation of $U_{d}$, $V_{d}$, $S_{d}$, respectively. The following rewrite the expression for message propagation at each layer of the reconfigured user–item interaction graph:

(8) $${\hat{e}}_{u_{i,k}}^{R}=\sigma \left(p\left({\hat{A}}_{SVD_{i,:}}\right)\cdot E_{v_{k-1}}\right)=\sigma \left(p\left({\left({\hat{U}}_{d}{\hat{S}}_{d}{\hat{V}}_{d}^{T}\right)}_{i,:}\right)\cdot E_{v_{k-1}}\right)$$

(9) $${\hat{e}}_{v_{j,k}}^{R}=\sigma \left(p\left({\hat{A}}_{SVD_{:,j}}^{T}\right)\cdot E_{u_{k-1}}\right)=\sigma \left(p\left({\left({\hat{V}}_{d}{\hat{S}}_{d}{\hat{U}}_{d}^{T}\right)}_{:,j}\right)\cdot E_{u_{k-1}}\right)$$

The final embedding representation of a node after reconstruction is the sum of all layer embedding representations:

(10) $$e_{u_{i}}^{R}=\sum_{k=0}^{K}{\hat{e}}_{u_{i,k}}^{R},e_{v_{j}}^{R}=\sum_{k=0}^{K}{\hat{e}}_{v_{j,k}}^{R}$$

#### 4.2.3. User–Device Similarity Modeling

We believe that there is some similarity in the TAP rules owned by different users with the same number of devices. For example, if user *a* owns three devices, a light bulb, camera, and smart socket, and user *b* owns three devices, a camera, light bulb, and leakage detector, then user *a* and user *b* may have the same TAP rule: if Camera detects a person, then turn on the Light. Based on this, we obtain a user–user similarity embedding matrix that reflects the number of devices owned by each user. The procedure is as follows. Firstly, we construct a matrix of users and the number of devices that they own: $e_{device}\in R^{m\times c}$, where *m* is the number of users, *c* is the total number of device types, and each row in the matrix $e_{device}$ represents the number of devices of each type owned by each user. Secondly, the matrix $e_{device}$ is normalized to obtain the matrix $A_{device}$, and the user–user similarity embedding matrix is obtained by the following expression:

(11) $$A_{uu}=A_{device}\cdot A_{device}^{T}$$

(12) $$e_{uu}=AGG\left(A_{uu}\right)$$

where $A_{uu}$ is the similarity matrix, each value represents the cosine similarity between users, and AGG(·) is an aggregation function in the GCN.

#### 4.2.4. Graph Contrastive Learning

In our approach, graph contrastive learning involves two main parts. The first part is to contrast the SVD-enhanced view embedding with the original view embedding in the InfoNCE loss:

(13) $$L_{u_{s}}=\sum_{i=0}^{I}\sum_{k=0}^{K}-\log\frac{\exp\left(s\left({\hat{e}}_{u_{i,k}},{\hat{e}}_{u_{i,k}}^{R}\right)/t\right)}{\sum_{i\prime =0}^{I}\exp\left(s\left({\hat{e}}_{u_{i,k}},{\hat{e}}_{u_{i\prime ,k}}^{R}\right)/t\right)}$$

where *t* is the temperature coefficient. Similarly, the InfoNCE loss of item $L_{v_{s}}$ can be obtained. The second part is to compare the user–user similarity view embedding with the original view embedding in the InfoNCE loss:

(14) $$L_{d}=\sum_{i=0}^{I}\sum_{k=0}^{K}-\log\frac{\exp\left(s\left({\hat{e}}_{u_{i,k}},e_{uu_{i,k}}\right)/t\right)}{\sum_{i\prime =0}^{I}\exp\left(s\left({\hat{e}}_{u_{i,k}},e_{uu_{i\prime ,k}}\right)/t\right)}$$

Finally, we jointly optimize the two-part contrastive loss with the objective function of the recommendation task:

(15) $$L=L_{rec}+\mu_{1}\cdot \left(L_{u_{s}}+L_{v_{s}}\right)+\mu_{2}\cdot L_{d}$$

where $L_{rec}=\sum_{i=0}^{I}\sum_{s=1}^{S}max\left(0,1-{\hat{y}}_{i,pos_{s}}+{\hat{y}}_{i,neg_{s}}\right)$, ${\hat{y}}_{i,pos_{s}}$ and ${\hat{y}}_{i,neg_{s}}$ denote the prediction scores of a pair of positive and negative items for user i, respectively.

The source code of our model is available at https://github.com/609bob/GCL4TAP (Accessed on 30 June 2024).

## 5. Experimental Evaluation

### 5.1. Experimental Settings

#### 5.1.1. Dataset

The Wyze dataset is a large-scale, user-centric dataset explicitly designed for smart home device rule recommendation research [25]. The dataset was created by Wyze Labs. It contains more than 1 million smart home device rules collected from 300,000 different users of Wyze Labs, providing extensive and diverse real-world data for today’s smart home device rule recommendation research. The original intention when creating the Wyze dataset was to address the challenges of cross-device learning in smart device rule recommendation (i.e., the lack of comprehensive and scalable datasets) and tailor it so it had many users with heterogeneous data. This dataset helps to develop and evaluate personalized rule recommendation algorithms to achieve smart home process automation while respecting user privacy.

The Wyze dataset covers a total of 16 common smart home devices in everyday life, and there are multiple instances of each device type. For example, a bedroom light and a hallway light, although both belonging to the category of lights, are different instances, so these two types of lights may have different combinations of connections, which can lead to a situation whereby the same type of device may have different rules. The dataset consists of two files, rule.csv and device.csv, which provide information about the rules controlling the behavior of Wyze smart home devices and user-owned devices. The rules in the dataset are simplified into the following structure: <trigger entity, trigger–action pairs, action entity>, where the trigger–action pair represents the type of connection established between different devices. For example, a rule such as “connecting a smart doorbell to a camera, pressing the doorbell will trigger the camera to turn on and record” can be represented as <doorbell, doorbell–press–turn-on–record, camera>; it contains 1323 unique trigger–action pairs, which can result in 2968 unique rules.

In the experiments described in this article, we selected 70,000 rules on the Wyze dataset. We filtered out the data where users had less than 3 rules, resulting in 3512 users, 2732 unique rules, and 24,236 interactions. We stored the user rules in the form of “user–items”, designating the user as a user and the rules owned by the user as items. The details of the dataset used for the experiments are shown in the following Table 3.

#### 5.1.2. Compared Methods

To validate the performance of our proposed framework, we select the following state-of-the-art recommendation algorithms for comparison.

**Light GCN [26]:** This model is a simplified and more efficient version of the graph convolutional network for recommendation systems. It removes two fundamental components of traditional graph convolutional networks—feature transformation and nonlinear activation—while only retaining the domain aggregation and hierarchical combination modules. The embedding representations of users and items are learned by linear propagation on top of the user–item interaction graph, and then the learned embeddings from all layers are weighted and summed to obtain the final representation.

**simGCL [17]:** This method eliminates the graph augmentation process commonly used in traditional graph contrastive learning. Instead, it generates contrastive views by introducing uniform noise into the embedding space, optimizing the contrastive loss to enhance the model performance. By smoothly adjusting the uniformity of the learned representations, this approach surpasses graph augmentation-based contrastive learning methods in both recommendation accuracy and training efficiency.

**LR-GCCF [27]:** This method is based on graph convolution networks and aims to solve the issues of traditional graph convolution network-based recommendation models facing training difficulties and over-smoothing problems. The approach removes nonlinear activation and introduces a residual network structure to improve the recommendation performance. In this way, the model is both easy to train and scales well to large datasets.

**NGCF [28]:** This method exploits the user–item graph structure by propagating embeddings over the graph structure. This can effectively encode higher-order connectivity relations into the embedding, explicitly injecting collaborative signals into the embedding process. This approach not only enhances the quality of the embeddings but also significantly improves the accuracy and effectiveness of predictions in recommendation systems.

**LightGCL [29]:** This is a lightweight yet highly effective graph contrastive learning (GCL) framework for recommendation systems. It leverages singular value decomposition (SVD) to guide graph augmentation, extracting valuable insights from user–item interactions while incorporating the global collaborative context into the representation of the augmented view. By doing so, it preserves both user-specific preferences and cross-user global dependencies. The augmented view, generated through SVD, is then contrasted with the original view. This approach not only enhances the training efficiency but also alleviates issues related to inaccurate comparison signals found in existing GCL methods by integrating global collaborative relationships.

#### 5.1.3. Evaluation Metrics

To evaluate the recommendation performance of our proposed GCL4TAP, we utilize three commonly used evaluation metrics in recommendation systems. The definition of each metric is shown below.

**HR@k:** The hit rate (HR) denotes the percentage of the number of rules containing rules that the user actually has in the top-*k* list of rule recommendations to the total number of rules that the user actually has, and the formula is shown below:

(16) $$HR@k=\frac{\left|Q_{hit}^{k}\right|}{\left|Q_{all}\right|}$$

where $\left|Q_{hit}^{k}\right|$ denotes the sum of the number of rules that a user actually has in the first *k* results recommended; $\left|Q_{all}\right|$ denotes the total number of rules that all users actually have.

**NDCG@k:** The normalized discounted cumulative gain (NDCG) is a metric for the evaluation of the performance of an information retrieval system and is particularly useful in evaluating the quality of result ordering in a search engine or recommendation system. It combines the relevance of the results and the positions of the results to measure the overall quality of the top *k* results that users care about most. Its calculation formula is shown below:

(17) $$NDCG@k=\frac{DCG@k}{IDCG@k}$$

where DCG@k denotes the cumulative discounted gain, which is calculated by weighting and summing the correlations of the results according to the ranked positions, as shown in the following formula:

(18) $$DCG@k=\sum_{i=1}^{k}\frac{2^{rel_{i}}-1}{\log_{2}\left(i+1\right)}$$

where $rel_{i}$ is the relevance score of the *i*-th result in the recommended results, which, in our experiments, takes only the values 0 and 1. If the result in the recommended list is not a real rule owned by the user, it takes the value 0; conversely, it takes the value 1; and *k* denotes the top-*k* recommended results. IDCG@k denotes the ideal cumulative discount gain, which is the optimal DCG value after sorting the results according to the correlation, and it is calculated as shown below:

(19) $$IDCG@k=\sum_{i=1}^{\left|REL\right|}\frac{2^{rel_{i}}-1}{\log_{2}\left(i+1\right)}$$

where $\left|REL\right|$ denotes the first *k* results in the list of recommended results in the ideal case.

**AUC:** The area under the ROC (AUC) indicates the overall performance of the rule recommendation model. It is a widely used metric in machine learning evaluations. The higher the AUC, the better the model’s ability to recommend.

#### 5.1.4. Parameter Settings

For the comparison algorithms employed in this study, we use the default parameters used in the original literature and do not adjust the hyperparameters. In our proposed algorithm, the embedding dimension *d* is tuned within {16,32,64,128,256}, and the optimal parameter 128 is chosen. The batch size is set to 256. Two convolutional layers are used for the GCN model. The regularization coefficients $\mu_{1},\mu_{2}$ are tuned within {0.01,0.05,0.1,0.2,0.3}, and finally $\mu_{1}$ is set to 0.1 and $\mu_{2}$ is set to 0.05. The learning rate lr is tuned within {$1\times 10^{-5}$, $1\times 10^{-4}$, $5\times 10^{-4}$, $1\times 10^{-3}$, $1\times 5^{-3}$} and finally determined to be $1\times 10^{-3}$. The temperature parameter *t* is set to 0.2. The rank *d* of the SVD is set to 5.

### 5.2. Experimental Results

In this part, we randomly divide the dataset into an 80% training set and 20% test set. Three performance metrics, HitRate@k, NDCG@k, and AUC, are used to evaluate the performance of our model. The same dataset and evaluation metrics are used to test the five comparison algorithms introduced in the previous section to verify the performance of our proposed model. The experimental results are recorded in Table 4. From the table, we can summarize the following important information:

1. The performance of GCL4TAP is significantly better than that of the other five compared methods in all three metrics: HitRate@k, NDCG@k, and AUC;
2. Compared with the LightGCL method, the performance of GCL4TAP is improved by 4.41%, 1.58%, and 2.96% in the three metrics of HitRate@50, NDCG@50, and AUC, respectively.

### 5.3. Ablation Study

We performed ablation experiments on the components of GCL4TAP to illustrate the effectiveness of the improved parts that we added. The experimental results are shown in Table 5. GCL4TAP-r denotes that the model contains only the recommendation loss for recommendation learning; GCL4TAP-r-s denotes that the model contains both the recommendation loss and the SVD contrastive loss; GCL4TAP-r-d denotes that the model includes both the recommendation loss and the device relevance contrastive loss; and GCL4TAP denotes our model, which contains a total of three components, i.e., the recommendation loss, the SVD contrastive loss, and the device relevance contrastive loss. From Table 5, we can conclude that when the model contains only the recommendation loss (i.e., GCL4TAP-r), the performance is the worst; when the SVD loss is added (i.e., GCL4TAP-r-s), the performance of the model is improved, which indicates that the SVD loss is helpful in the training of the model. When the model adds only the device relevance loss (i.e., GCL4TAP-r-s), the performance is improved, which indicates that the SVD loss is helpful in the training of the model. For GCL4TAP-r-d, The performance of the model is improved compared to that of GCL4TAP-r. Nonetheless, the performance improvement in the two metrics of HitRate@k and AUC is higher than that of GCL4TAP-r-s, and the improvement in NDCG@k is not as high as that of GCL4TAP-r-s. Relative to GCL4TAP-r-s, when the two-part contrastive loss is added (i.e., GCL4TAP), the performance of the model is significantly improved over GCL4TAP-r. In summary, the model’s SVD contrastive loss and the device correlation contrastive loss are vital in enhancing the model’s performance.

### 5.4. Parameter Sensitivity Analysis

We performed a sensitivity analysis on the hyperparameters $\mu_{1}$, $\mu_{2}$, the embedding dimension *d*, the learning rate lr, and epoch, respectively, and the results are shown below.

**Figure 4:**
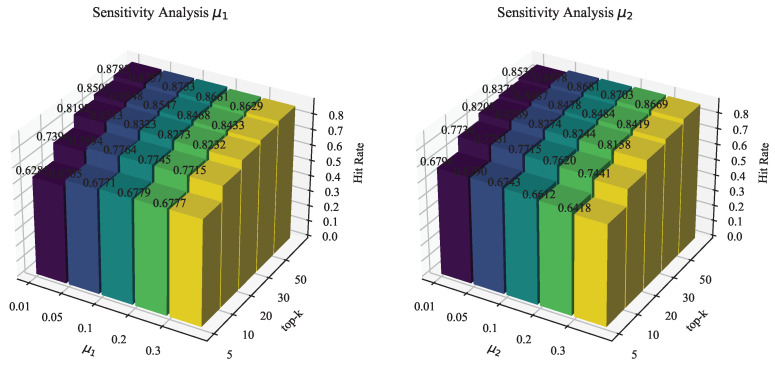
Sensitivity analysis of $\mu_{1}$, $\mu_{2}$.

Figure 4 shows the performance in terms of the hit rate on each top-*k* when the two parameters $\mu_{1},\mu_{2}$ are taken in {0.01,0.05,0.1,0.2,0.3}, respectively. From the left of Figure 4, it is easy to see that when the value of $\mu_{1}$ is taken as 0.1, the overall performance in terms of the hit rate on the top-*k* is the best; according to the right of Figure 4, when the value of $\mu_{2}$ is taken as 0.05, the overall performance in terms of the hit rate is the best.

**Figure 5:**
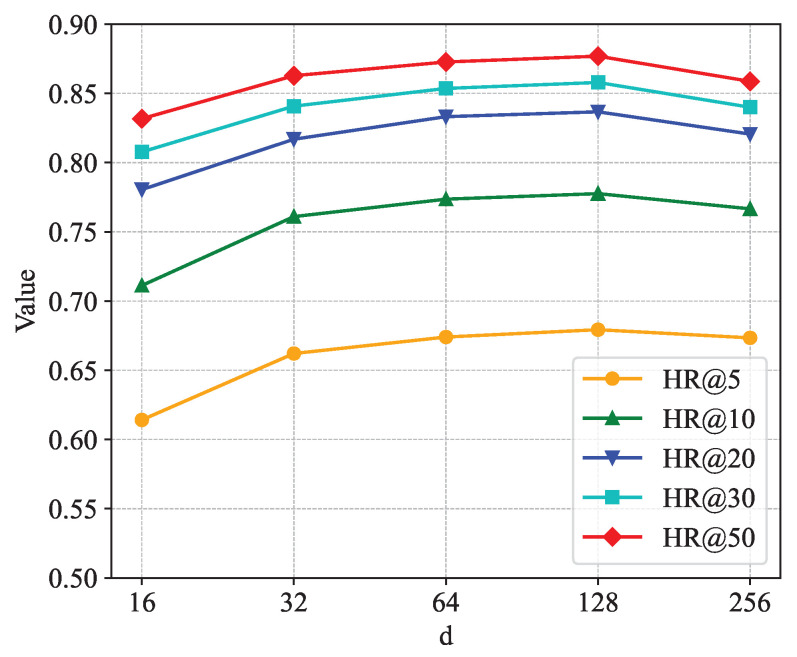
Impact of dimension on hit rate.

**Figure 6:**
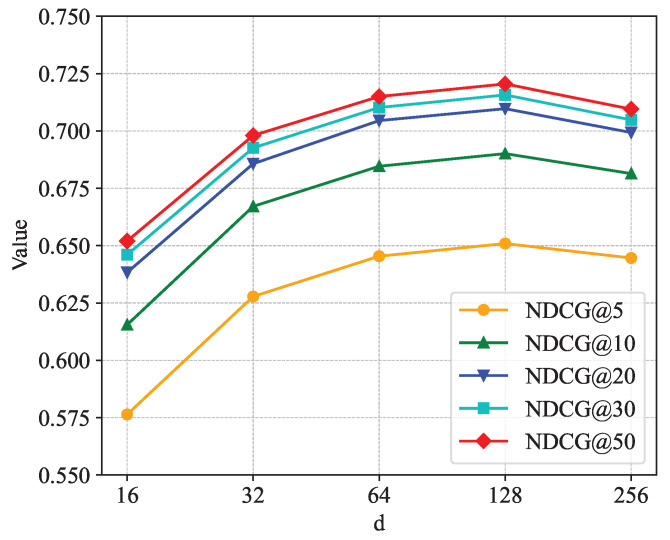
Impact of dimension on NDCG.

**Figure 7:**
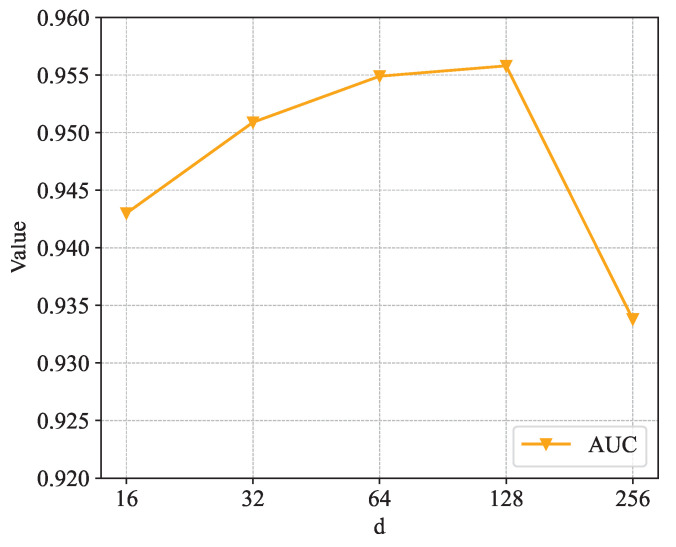
Impact of dimension on AUC.

Figure 5, Figure 6 and Figure 7 show the sensitivity analysis of the embedding dimension parameter *d*. Figure 5 shows the changes in HR@5, HR@10, HR@20, HR@30, and HR@50 in different embedding dimensions. It is not difficult to see from the figure that each curve rises as *d* increases. When *d* = 128, each curve rises to the highest point; when the value of *d* continues to increase, each curve shows a downward trend. Figure 6 shows the changes in NDCG@5, NDCG@10, NDCG@20, NDCG@30, and NDCG@50 in different embedding dimensions. The trend of the NDCG is similar to that of the hit rate, and each curve rises to the highest point at *d* = 128. Figure 7 shows the changes in the AUC in different embedding dimensions, and, as the value of *d* increases, the curve continuously rises, and the AUC takes the largest value when *d* = 128. In summary, when the embedding dimension parameter *d* is taken as 128, the performance of the model is the best.

**Figure 8:**
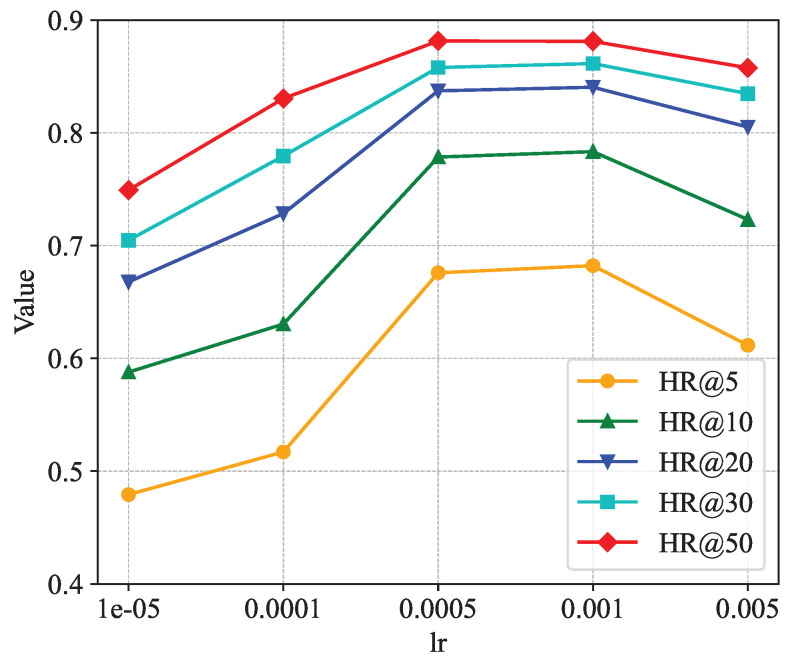
Impact of learning rate on hit rate.

**Figure 9:**
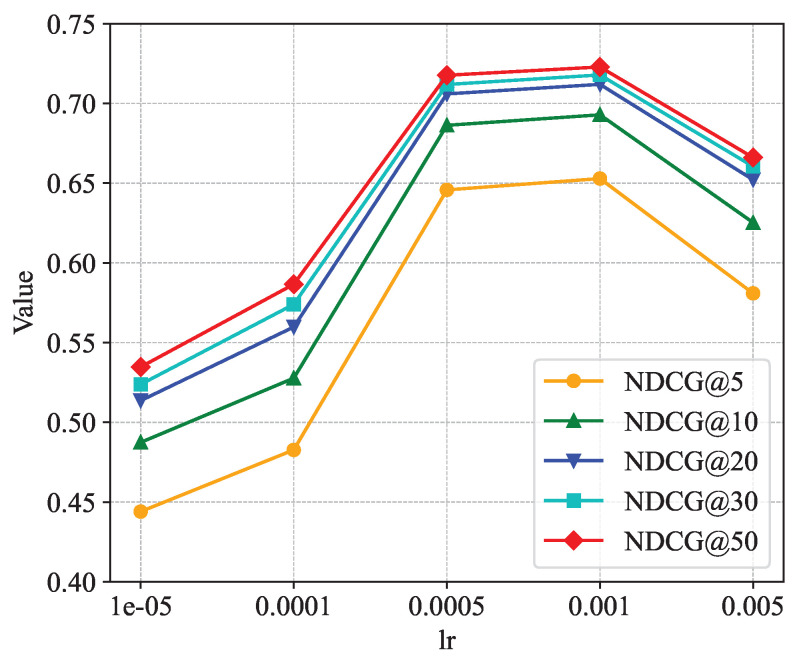
Impact of learning rate on NDCG.

**Figure 10:**
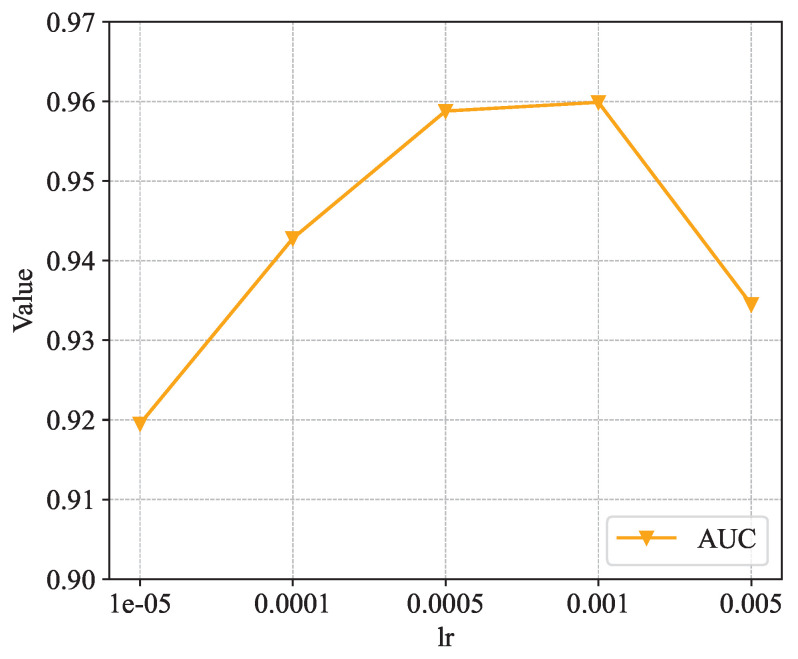
Impact of learning rate on AUC.

Figure 8, Figure 9 and Figure 10 show the analysis of the sensitivity of the learning rate. The three figures show the trends of the Hit Rate@k, NDCG@k, and AUC metrics under different learning rates, respectively. From the figures, it can be found that when the learning rate parameter lr is taken as 0.0005 or 0.001, the values of all indicators are roughly equal and all of them reach the maximum. Thus, the two learning rates are roughly equivalent to the model’s recommendation performance, and, in our model, the learning rate lr is taken as 0.001.

**Figure 11:**
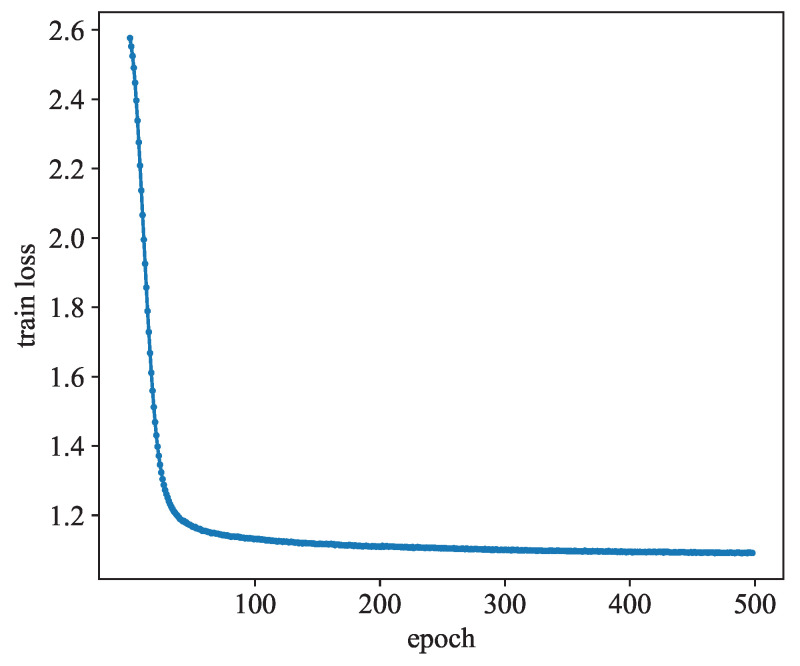
Impact of epoch on train loss.

Figure 11 shows the trend of the model training loss, from which we can see that as the number of epochs increases, the training loss of the model will converge to a smaller value. In our model, the epoch is taken as 500.

### 5.5. Discussion

In this section, we examine the conclusions obtained in Section 5.2 and give some of our own reflections on the results. The first conclusion shows that our proposed GCL4TAP method is more efficient in rule recommendation compared to some existing mainstream recommendation methods. The reason is that the GCL4TAP method combines the advantages of graph convolutional networks and graph contrastive learning. It uses graph convolutional networks to fully explore the association information between users and rules and uses graph contrastive learning methods to fully consider the device similarity. Regarding the second conclusion, the reason that the GCL4TAP method has a significant improvement in the three metrics compared to LightGCL is that we add the user’s device information to the model so that the model can analyze the similarity between the devices owned by the user. The data show that the device similarity module that we added enables an improvement in the model’s performance. Although our proposed GCL4TAP method demonstrates significant improvements compared to several existing mainstream recommendation approaches, we have identified certain limitations during our experiments. For instance, when dealing with large-scale datasets, our method exhibits relatively slow processing speeds, causing each experiment to take several hours. Therefore, optimizing the efficiency and enhancing the runtime performance of the GCL4TAP algorithm will be the key focus of our future research.

## 6. Conclusions

The number of IoT devices has significantly increased in recent years due to advances in communication technologies. This has led to growing potential for the pairing and combination of devices. For non-specialized users, this vast combination space makes it challenging to automate IoT devices by creating IF-THEN rules. To address this challenge, this article proposes a rule recommendation framework called GCL4TAP. The framework predicts user preferences for TAP rules, enabling personalized rule recommendations. In GCL4TAP, we first use a data partitioning method called DATA2DIV to establish cross-user rule relationships, represented as a user–rule bipartite graph. Then, we determine the similarity between users based on the categories and quantities of devices that they own, represented as a user–user graph. Finally, these graphs are transformed into low-dimensional vector representations of the users and rules using graph contrastive learning techniques, providing efficient and accurate TAP rule recommendations. We then compare the performance of our proposed framework with existing recommendation algorithms on the Wyze dataset. The experimental results show that our framework significantly outperforms the comparison algorithms in terms of the Hit Rate@k, NDCG@k, and AUC metrics.

## Figures and Tables

**Table 1:** The frequently used notations.

Notation	Explanation	Dimension
$L_{gcl}$	InfoNCE loss	-
$e_{i}$	Embedding representation of the original view	1×n
$e_{u_{i}}$	Embedding representation of user *i*	1×D
$e_{v_{j}}$	Embedding representation of rule *j*	1×D
$E_{u}$	Embedding representation of all users	m×D
$E_{v}$	Embedding representation of all rules	n×D
${\hat{e}}_{u_{i,k}}$	Aggregate embedding representation of user *i* in *k*-th layer	1×D
${\hat{e}}_{v_{j,k}}$	Aggregate embedding representation of rule *j* in *k*-th layer	1×D
$\hat{A}$	Normalized adjacency matrix	m×n
σ(·)	Activation function	-
$u_{i}$	User *i*	-
$v_{j}$	Rule *j*	-
*U*	Eigenvectors of the correlation matrix between rows of the normalized adjacency matrix	m×m
*V*	Eigenvectors of the correlation matrix between columns of the normalized adjacency matrix	n×n
*S*	Diagonal array storing singular values of adjacency matrix	m×n
$U_{d}$	First *d* rows of *U*	m×d
$V_{d}$	First *d* rows of *V*	n×d
$S_{d}$	Diagonal matrix corresponding to the first d largest singular values	d×d

**Table 2:** Structure and composition of rules in the Wyze dataset.

Component	Trigger	Action	Rule
	**Device** **Function**	**Device** **Function**	**Description**
Rule of Figure 1a	LeakSensor Detect a leak	LightStrip Blink	If LeakSensor detects a leak, then LightStrip’s color blinks
Rule of Figure 1b	Camera Detect a person	Light Turn on	If Camera detects a person, then turn on the Light

**Table 3:** Details of the dataset used for the experiment.

Dataset	User	Item	Interaction
Wyze	3512	2732	24,236

**Table 4:** Results of the experiment.

Method	LightGCN	SimGCL	LR-GCCF	NGCF	LightGCL	GCL4TAP
HR@5	0.5650	0.4156	0.6141	0.5370	0.6793	**0.6830**
HR@10	0.6895	0.4913	0.7236	0.6512	0.7675	**0.7837**
HR@20	0.7709	0.5606	0.8034	0.7651	0.8119	**0.8395**
HR@30	0.8087	0.5955	0.8343	0.8132	0.8292	**0.8618**
HR@50	0.8425	0.6376	0.8629	0.8599	0.8454	**0.8827**
NDCG@5	0.5233	0.4172	0.4633	0.3056	0.6521	**0.6523**
NDCG@10	0.5735	0.4480	0.5007	0.3542	0.6870	**0.6921**
NDCG@20	0.6004	0.4702	0.5220	0.3962	0.7019	**0.7106**
NDCG@30	0.6111	0.4793	0.5290	0.4139	0.7069	**0.7169**
NDCG@50	0.6196	0.4890	0.5348	0.4307	0.7109	**0.7221**
AUC	0.9479	0.8948	0.8054	0.9412	0.9316	**0.9592**

**Table 5:** Results of ablation experiments.

Variant	GCL4TAP-r	GCL4TAP-r-s	GCL4TAP-r-d	GCL4TAP
HR@5	0.6496	0.6793	0.6504	**0.6830**
HR@10	0.7363	0.7675	0.7474	**0.7837**
HR@20	0.7938	0.8119	0.8195	**0.8395**
HR@30	0.8172	0.8292	0.8471	**0.8618**
HR@50	0.8393	0.8454	0.8759	**0.8827**
NDCG@5	0.6108	0.6521	0.6120	**0.6523**
NDCG@10	0.6456	0.6870	0.6504	**0.6921**
NDCG@20	0.6647	0.7019	0.6742	**0.7106**
NDCG@30	0.6714	0.7069	0.6890	**0.7169**
NDCG@50	0.6769	0.7109	0.6890	**0.7221**
AUC	0.9351	0.9316	0.9558	**0.9592**

## Data Availability

Data are contained within the article.
